# Increase of Reproductive Life Span Delays Age of Onset of Parkinson’s Disease

**DOI:** 10.3389/fneur.2017.00397

**Published:** 2017-08-21

**Authors:** Dominik Frentzel, Grigorij Judanin, Olga Borozdina, Jochen Klucken, Jürgen Winkler, Johannes C. M. Schlachetzki

**Affiliations:** ^1^Department of Molecular Neurology, University Hospital Erlangen, Friedrich-Alexander Universität (FAU) Erlangen-Nürnberg, Erlangen, Germany; ^2^Department of Applied Econometrics and International Political Economy, Goethe University Frankfurt, Frankfurt, Germany; ^3^Department of Cellular and Molecular Medicine, University of California, San Diego, La Jolla, CA, United States

**Keywords:** Parkinson’s disease, gender, estrogen status, disease onset, dopamine

## Abstract

One striking observation in Parkinson’s disease (PD) is the remarkable gender difference in incidence and prevalence of the disease. Data on gender differences with regard to disease onset, motor and non-motor symptoms, and dopaminergic medication are limited. Furthermore, whether estrogen status affects disease onset and progression of PD is controversially discussed. In this retrospective single center study, we extracted clinical data of 226 ambulatory PD patients and compared age of disease onset, disease stage, motor impairment, non-motor symptoms, and dopaminergic medication between genders. We applied a matched-pairs design to adjust for age and disease duration. To determine the effect of estrogen-related reproductive factors including number of children, age at menarche, and menopause on the age of onset, we applied a standardized questionnaire and performed a regression analysis. The male to female ratio in the present PD cohort was 1.9:1 (147 men vs. 79 women). Male patients showed increased motor impairment than female patients. The levodopa equivalent daily dose was increased by 18.9% in male patients compared to female patients. Matched-pairs analysis confirmed the increased dose of dopaminergic medication in male patients. No differences were observed in age of onset, type of medication, and non-motor symptoms between both groups. Female reproductive factors including number of children, age at menarche, and age at menopause were positively associated with a delay of disease onset up to 30 months. The disease-modifying role of estrogen-related outcome measures warrants further clinical and experimental studies targeting gender differences, specifically hormone-dependent pathways in PD.

## Introduction

Parkinson’s disease (PD) is a chronic, progressive neurodegenerative disease, and the most common movement disorder (1). The incidence and prevalence of PD in males substantially exceeds those of females (2, 3). Large meta-analyses of incidence studies determined 1.5–2 times higher risk for men to develop PD (2, 4, 5) and roughly 2-year earlier disease onset in men compared to women (4, 6).

The clinical phenotype of PD, which is defined by the presence of bradykinesia and at least one additional motor symptom including rigidity and resting tremor (7), may also differ between both genders. Tremor is more prevalent as initial symptom in women (6), whereas the progression rate of motor impairment and daily l-DOPA doses are increased by 15% in men (8, 9). Besides prototypical motor symptoms, a broad spectrum of non-motor symptoms such as obstipation, hyposmia, rapid eye movement sleep behavior disorder (RBD), depression, cognitive impairment, restless legs syndrome (RLS), and urinary dysfunction are highly prevalent in PD affecting quality of life and disease severity (10, 11). Hyposmia and RBD are more prevalent in men (12–15), whereas depression and anxiety are more prevalent in women (14, 16). In a Chinese PD cohort, female *de novo* PD patients showed more severe depressive symptoms than men (17).

Despite recent advances in deciphering the molecular pathogenesis and genetic predispositions in PD, the causal relationships underlying the observed gender differences are not well understood till date. *In vivo* studies of acute PD models (18–20) suggest a neuroprotective effect of the sex hormone estrogen, which may explain the differences in prevalence. This experimental evidence is further supported by epidemiological findings, such as (i) the age of PD onset correlates with the age of menopause, length of fertile life, and parity (6, 21, 22) and (ii) application of estrogen supplementation in postmenopausal women has been linked to a lower risk of developing PD (23, 24). Furthermore, estrogen supplementation improved motor function in patients with PD (25, 26). Genome-wide association studies also revealed common genetic variants in the estrogen-related gene PR domain 2 to an increased susceptibility for PD (27, 28).

The aim of this study is to explore gender differences in a German single center movement disorder outpatient clinic with regard to demographic characteristics, type and dosage of dopaminergic medication, and motor and non-motor symptoms in PD. Hence, we used our movement disorder center database to address (1) gender differences in dopaminergic medication and (2) the role of estrogen-related reproductive factors including number of children and reproductive life span on the age of disease onset.

## Materials and Methods

### Study Population

The study was approved by the Institutional Review Board for Ethics, FAU Erlangen-Nürnberg (152_15 Bc). We consecutively extracted the clinical data of 226 PD patients from our movement disorder center database between April 2010 and March 2015. Only patients diagnosed with PD according to the consensus criteria of the German Society of Neurology [analog to the National Institute of Neurological Disorders and Stroke diagnostic criteria for PD (29)] were included. Our database includes general demographic information such as age at presentation, sex, weight, height, body mass index (BMI) and a general dataset including diagnoses, medication, and family history. Disease staging was based on the Hoehn and Yahr (H&Y) Disability Scale (30). Motor impairment of PD subjects was assessed using the Unified Parkinson’s Disease Rating Scale (UPDRS) motor score part III rating (31). Type and dose of dopaminergic medication was compared using the levodopa equivalent daily dose (LEDD) calculated as previously described by Tomlinson et al. (32). We defined the age of disease onset as the year in which first signs of motor impairment were reported either by the patient or relatives. Non-motor symptoms were recorded using non-motor assessment scales (NMS) for PD (33), and patients were tested for the presence of depressive symptoms based on the Zung self-rating depression scale (34). We applied a matched-pairs design to assess the effect of gender on LEDD. Thus, each female patient was matched per age and disease duration to a male patient. This strategy enabled us to match 78 out of 79 female patients to male counterparts.

### Estrogen Status Questionnaire

To assess the estrogen status of our female PD population, we used a brief self-rating, standardized instrument based on previously published reports (6, 21, 35). Aim of the questionnaire was to determine (i) the number of children, (ii) the age at menarche, (iii) age at menopause (defined as the age 12 months after last menstrual cycle), and (iv) hormonal replacement therapy (HRT). The following questions were included in the questionnaire: (1) How many children do you have? (2) At what age did you have your first menstruation? (3) At what age did you have your last menstruation? (4) Have you received any HRT including estrogens?

The questionnaire was mailed to 79 female patients with a 96% return quote (76/79). For the analysis on whether the age at menopause and fertile life span influenced the age of disease onset, we focused on the period of endogenous estrogen exposure prior to the onset of PD. Therefore, women reporting menopause after PD onset (*n* = 15) or who had undergone hysterectomy (*n* = 7) were excluded.

### Statistical Analysis

Demographic characteristics, the age of onset and disease duration, and clinical rating scales were given as mean ± SD. Potential differences in these features between men and women were analyzed using a two-sample *t*-test. Normal distribution was ascertained by Kolmogorov–Smirnov test for all variables except UPDRS-III. For analysis of UPDRS-III, Mann–Whitney *U*-test was used accordingly. Non-motor symptoms were indicated as proportions and compared by a Chi-square statistics. LEDD was examined through univariate analyses of variances, with gender, H&Y disease staging, and BMI as independent variables. Type of dopaminergic medication was analyzed by means of different categories (l-DOPA only; l-DOPA + others; dopamine agonist only; dopamine agonist + others; l-DOPA + dopamine agonist; and others) and tested with Chi-square statistics.

Moreover, female reproductive data were given as mean ± SD and linear regression models were applied to demonstrate a relationship between hormonal and clinical parameters in PD. Consequently, the age of disease onset was regressed on age at menarche, age at menopause, number of children, and duration of fertile life. The correlation coefficient, standard error (SE), and beta were determined. The Pearson coefficient was calculated to quantify the correlation between the duration of fertile life and the age of PD onset. The application of hormone replacement therapy was not included as the sample size was too small (*n* = 11). All data were analyzed using IBM’s SPSS software (version 21.0). A value of *p* < 0.05 was set to be statistically significant.

## Results

To detect differences in the clinical phenotype of PD between women and men, we retrospectively analyzed the data of 226 patients of the Erlangen movement disorder database. As presented in Table 1, male patients outnumbered females in a ratio of 1.9:1 (147 vs. 79 patients, respectively) at baseline. Age was similar between both groups. Age of onset and disease duration did not significantly differ between both groups. Compared to females, male PD patients were more severely affected, as indicated by higher scores in H&Y (2.1 ± 1.0 vs. 1.8 ± 0.9, two-sample *t*-test, *p* < 0.05) and UPDRS-III scale (20.8 ± 12.2 vs. 17.4 ± 12.0, Mann–Whitney *U*-test, *p* < 0.05).

**Table 1:** Characteristics of Parkinson’s disease study population.

	Overall population *N* = 226		Gender-matched population *N* = 156	
**Sex**		
Male	147 (65.0%)		78 (50.0%)	
Female	79 (35.0%)		78 (50.0%)	
**Age (years)**		
Male	62.8 ± 11.1 [36–85]		62.6 ± 11.4 [38–85]	
Female	62.4 ± 11.7 [36–84]		62.4 ± 11.8 [36–84]	
**Body mass index**		
Male	26.3 ± 4.0 [19.0–42.2]		26.2 ± 3.7 [19.9–41.9]	
Female	25.6 ± 5.0 [18.3–49.2]		25.6 ± 5.1 [18.3–49.2]	
**Age of onset**		
Male	57.3 ± 11.1 [30–84]		57.4 ± 11.3 [30–84]	
Female	57.1 ± 11.8 [30–84]		57.1 ± 11.8 [30–84]	
**Disease duration**		
Male	6.0 ± 5.0 [0–24]		5.8 ± 4.8 [0–24]	
Female	5.8 ± 5.0 [0–26]		5.8 ± 5.0 [0–26]	
**Unified Parkinson’s Disease Rating Scale-III**		
Male	**20.8 ± 12.2 (*n* = 147) [2–85]**^b^		19.0 ± 10.0 (*n* = 78) [2–45]	
Female	**17.4 ± 12.0 (*n* = 77) [3–50]**^b^		17.6 ± 12.1 (*n* = 76) [3–50]	
**Hoehn and Yahr**		
Male	**2.1 ± 1.0 [1–5]**^a^		2.0 ± 0.9 [1–4]	
Female	**1.8 ± 0.9 [1–4]**^a^		1.8 ± 0.9 [1–4]	
**Zung**		
Male	47.3 ± 10.9 (*n* = 139) [0–75]		47.3 ± 9.8 (*n* = 72) [30–71]	
Female	48.3 ± 10.9 (*n* = 75) [28.8–80]		48.5 ± 10.8 (*n* = 74) [28.8–80]	

**Non-motor assessment scales**	**Male (%)**	**Female (%)**	**Male (%)**	**Female (%)**

Hyposmia	49.7	50.6	52.6	50.0
Obstipation	37.4	30.4	39.7	30.8
Urge symptoms	47.6	41.8	46.2	42.3
Nykturia	27.9	38.0	29.5	38.5
Rapid eye movement sleep behavior disorder	25.9	21.5	26.9	21.8
Restless legs syndrome	19.7	20.3	23.1	20.5
**Zung**				
Depression	39.5	45.3	40.2	45.9
**Levodopa equivalent daily dose**				
Male	**503.5 ± 462.7 [0–2076.0]**^c^		**438.4 ± 383.1 [0–1510.0]**^c^	
Female	**408.6 ± 374.8 [0–1387.0]**^c^		**413.8 ± 374.4 [0–1387.0]**^c^	

*Values in bold font indicate significant difference (p < 0.05) between male and female population. Range is given in brackets*.

*^a^Student’s t-test*.

*^b^Mann–Whitney U-test*.

*^c^Univariant analysis of variance*.

As non-motor symptoms have a profound impact on the quality of life in patients with PD, we screened for differences in hyposmia, obstipation, urge symptoms, depression, RBD, and RLS using self-rating questionnaires, but there were no significant differences between males and females. According to the NMS questionnaire (33), hyposmia was the most prevalent syndrome reported by ~50% of the PD patients and REM-sleep behavior disorder was reported by ~25%. Based on the Zung self-rating depression scale (34), 39.5% of male and 45.3% of the female PD patients showed signs of depression. Whereas 24.4% of male patients and 28.0% of female patients have mild depressive symptoms, 15.0% of male patients and 17.3% of female patients were identified with moderate-to-severe depression.

Next, we asked if the dose of dopaminergic medication differed between males and females in our cohort. We observed a significantly increased LEDD in men compared with women (503.5 ± 462.7 vs. 408.6 ± 374.8 mg; univariate analysis of variance, *F* = 133.79, *p* < 0.001, adjusted *R*^2^ = 0.540).

Regarding the type of dopaminergic medication, the majority of patients were taking dopamine agonists in combination with other drugs including l-DOPA, amantadine, monoamine oxidase type B inhibitors, or anticholinergics (men 27.2%, women 22.8%). Only 6.1% male patients and 10.1% female patients were treated with an l-DOPA monotherapy, whereas 14.1 and 16.5% received a combination of l-DOPA and a dopamine agonist, respectively (Table 2). We detected no gender difference in the type of dopaminergic medication.

**Table 2:** Type of dopaminergic medication of 226 Parkinson’s disease (PD) patients.

Type of PD medication	Male	Female	*p*-Value^a^
l-DOPA only	9 (6.1%)	8 (10.1%)	0.276
l-DOPA + other^b^	24 (16.3%)	13 (16.5%)	0.980
Dopamine agonist only	10 (6.8%)	1 (1.3%)	0.065
Dopamine agonist + other^b^	40 (27.2%)	18 (22.8%)	0.468
l-DOPA + dopamine agonist	27 (18.4%)	13 (16.5%)	0.720
Others^b^	36 (24.5%)	17 (21.5%)	0.615

*^a^Chi-square test*.

*^b^Others include amantadine, rasagiline, selegiline, biperidene, trihexyphenidyl, and bornaprine*.

Since the difference in the daily dose of dopaminergic medication may be due to a higher age and a longer disease duration in males in the present cohort, we matched the population based on gender, age, and disease duration to rule out their confounding effects, resulting in 78 matched gender PD patient pairs. We did not detect any significant gender differences in the mean motor score (UPDRS-III: 19.0 ± 10.0 for men vs. 17.6 ± 12.1 for women, Mann–Whitney *U*-test, *p* = 0.411) and the disease stage (H&Y: 2.0 ± 0.9 in men vs. 1.8 ± 0.9 in women, two-sample *t*-test, *p* = 0.374; Table 1). Nevertheless, LEDD values were still significantly increased in men (438.4 ± 383.1 mg men vs. 413.8 ± 374.4 mg women, univariate analysis of variance, *p* < 0.001). When integrating further factors into the LEDD model (univariate analysis of variance, *p* < 0.001, adjusted *R*^2^ = 0.712), H&Y had a very profound effect (univariate analysis of variance, *F* = 20.8, *p* < 0.001, η*p*^2^ = 0.308). Despite taking H&Y staging as one of the most relevant confounding factors into account, gender remained to be a significant and crucial factor for LEDD (univariate analysis of variance, *F* = 4.6, *p* < 0.05, η*p*^2^ = 0.032). The BMI did not have a significant influence on LEDD (univariate analysis of variance, *p* = 0.457). Applying the matched-pairs design, we did not observe significant differences in the type of dopaminergic medication between both genders (Table S1 in Supplementary Material).

**Figure 1:**
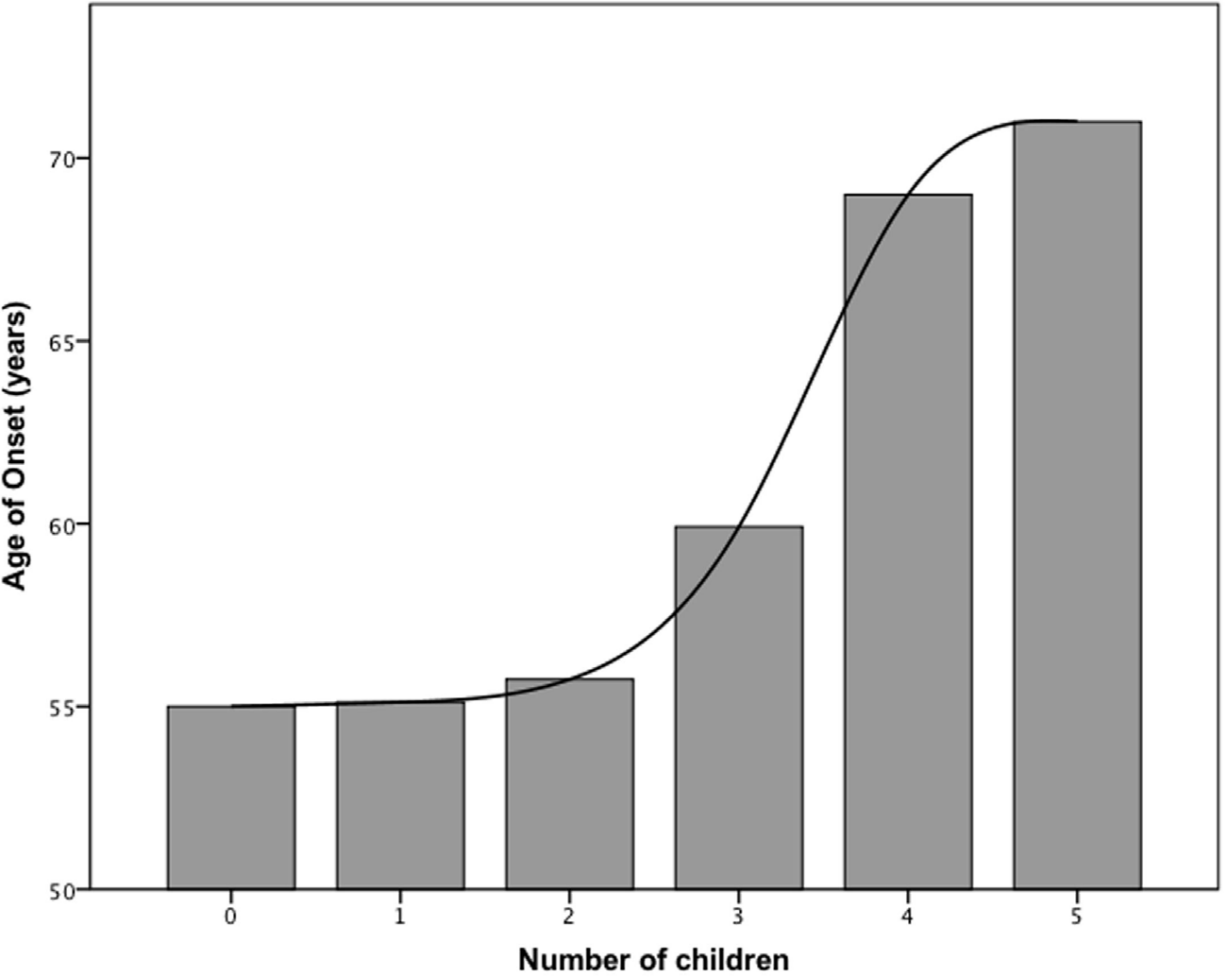
Bar graph showing the relationship between age of disease onset and number of children in females.

We then asked, whether estrogen status in female patients influenced the age of PD onset. To answer this question, we determined the number of children, the age of menarche, and the age of menopause of our female study population based on the applied questionnaire and conducted a linear regression analysis (Table 3). The age of disease onset correlated significantly with the number of children (linear regression analysis, beta = 0.229, *p* < 0.05, adjusted *R*^2^ = 0.053), i.e., birth of one child increased the age of disease onset by 2.6 years (Figure 1).

**Table 3:** Female Parkinson’s disease (PD) study population.

		Age of onset
Independent variables		Coefficient (SE) [*p*-value]
Number of children	1.8 ± 1.0 (*n* = 76) [0–5]	**2.64 (1.30) [<0.05]**
Age at menarche (years)	13.7 ± 1.7 (*n* = 76) [10–18]	0.88 (0.81) [0.283]
Age at menopause (years)^a^	50.5 ± 4.6 (*n* = 54) [37–61]	**0.73 (0.25) [<0.01]**
Reproductive life span^a^	36.9 ± 4.7 (*n* = 54) [25–46]	**0.62 (0.25) [<0.05]**

*Linear regression analysis of reproductive factors and age of PD onset. Values in bold font indicate significant relationship (p < 0.05)*.

*^a^Patients reporting PD onset before menopause or surgical menopause were excluded*.

*Coefficient: change in the dependent variable*.

**Figure 2:**
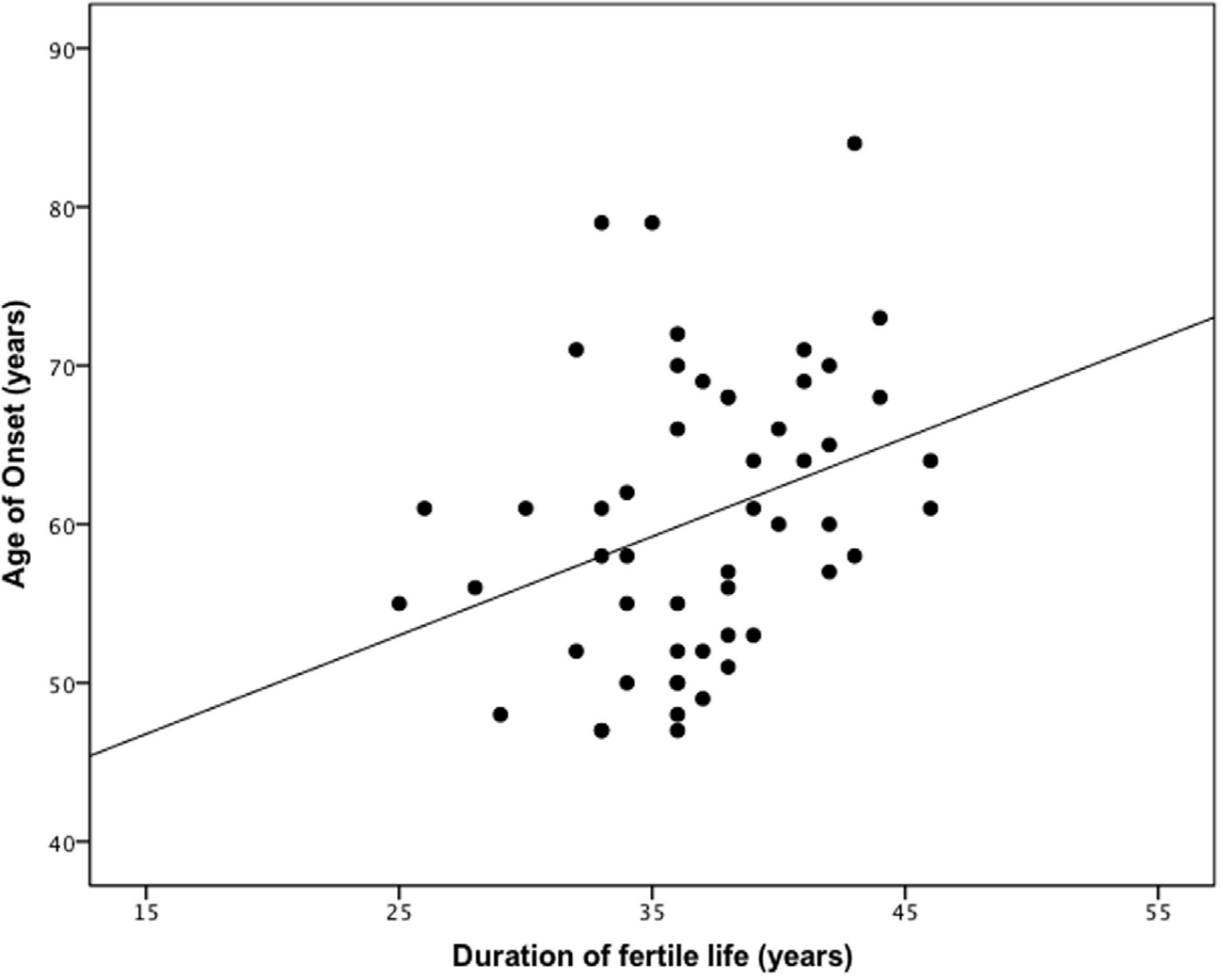
The duration of reproductive life influences the age of onset in female patients with Parkinson’s disease (PD). One year of prolonged fertility increases age of PD onset by 0.7 years. Pearson coefficient = 0.343 (*p* < 0.05).

The mean age at menarche and menopause was 13.7 and 50.5 years, respectively, with a mean duration of fertile life of 36.8 years (Table 3). The age at menopause (linear regression analysis, beta = 0.370, *p* < 0.01, adjusted *R*^2^ = 0.121) and duration of fertile life (linear regression analysis, beta = 0.343, *p* < 0.05, adjusted *R*^2^ = 0.117) were associated with the delayed disease onset. Specifically, 1 year of prolonged fertility increased age of PD onset by 0.7 years (Figure 2). We did not observe effects of the abovementioned reproductive factors on LEDD or UPDRS.

## Discussion

The present cross-sectional, retrospective single center study supports the general epidemiological observation of apparent gender differences in PD (2, 4–6). Interestingly, estrogen status in females had a major impact on the age of PD onset. We show that LEDD is lower in women when compared to men. However, we did not observe gender differences in the type of dopaminergic medication and clinical presentation of non-motor symptoms.

We included 226 PD patients from our movement disorder center database, resulting in a male to female ratio of 1.9:1. The gender ratio in the present cohort with a male predominance in PD is in line with previously published studies. An incidence study by Van den Eeden et al. revealed a 91% higher rate for men to develop PD in a Northern California cohort (2). In a meta-analysis of seven incidence studies (with the population size ranging from 182,934 to 3,869,162), Wooten et al. determined a weighted mean male to female ratio of 1.5 (5). Furthermore, Twelves et al. reviewed and identified a significantly greater ratio of males to females (1.5:2.0) in five of the nine incidence studies (4).

We observed significantly increased motor impairment and disease severity as measured by UPDRS-III and H&Y in male PD patients. However, differences regarding motor impairment and disease stage were not evident after the populations were matched to gender, age, and disease duration. The LEDD was overall lower in female patients, after controlling further for disease severity and BMI. Nyholm et al. (9) also described that men with PD required higher mean daily doses of l-DOPA, specifically by 17.4% (9). This finding was based on a nationwide study from Sweden conducted in 2007, in which 33,534 l-DOPA prescriptions from a drug register were analyzed and daily l-DOPA doses estimated for each patient, regardless of confirmed PD diagnosis, disease duration or body weight. Lyons et al. (8) reviewed 630 patients from a PD registry and controlled them for age and disease duration (8) and reported an increase in the mean daily l-DOPA dosages by 15.2% in men. This gender difference was even more prominent in advanced disease stages with disease duration greater than 5 years. Umeh et al. (36) detected no sex differences in type and dose of dopaminergic medications used in early PD (36). In this large multicenter study from the United States and Canada, 1,741 patients were registered within 5 years of PD diagnosis and adjusted for disease duration, motor impairment, and body weight. The study also employed LEDD instead of solely l-DOPA as a more appropriate overall measure for dopaminergic treatment. Of note, we did not observe that the BMI significantly interacts with the dosage of dopaminergic medication, confirming a previously published study (36).

We detected no gender differences in the presentation of non-motor symptoms. Approximately 40% of the male patients and 45% of the female patients in the present study fulfilled the criteria for depression, indicating a slight but not significant difference. Although two studies identified the female gender as a risk factor for depression in PD (16, 37), Tandberg et al. (38) described no difference between male and female PD patients (38). The mean frequency of depression in PD patients varies considerably, ranging from 22 to 46% (38–40), depending on the population and the method of diagnosis. While 50% of the patients reported olfactory impairment in the self-assessment of the NMS, hyposmia is generally described to be more common among PD patients (15). There is evidence that one-third of the patients with olfactory loss are not aware of the condition (41), and thus the NMS questionnaire is prone to underestimate the frequency of hyposmia in PD (42).

The role of estrogen and its relevance to PD is not well understood. Our findings indicate that endogenous estrogen affects age of disease onset. Number of children, age of menopause, and reproductive life span were associated with a delayed age of disease onset. Thus, increased circulating estrogen levels throughout a woman’s life delay the onset of PD supporting previous cross-sectional studies (6, 21, 22). Haaxma et al. (6) prospectively studied a cohort from the Netherlands and observed an increase of PD onset by 2.7 years per child and by 0.5 years per additional year of fertility (6). A case–control study further associated a shorter reproductive life span with an increased risk of developing PD (35). It should be noted that prospective cohort studies (43, 44) and case–control studies (45, 46) showed no significant relation between reproductive factors and PD risk. Our data support the observation that endogenous estrogen may be a protective factor for women against developing PD. The impact of exogenous estrogen on PD and its role as a disease-modifying hormone is similarly controversial. Postmenopausal hormonal therapy is reported to either improve motor function (25, 26) or to be without an effect on motor symptoms (47, 48). Moreover, estrogen level was associated with a decreased (23), increased (46), and equal risk to develop PD (43, 49).

The inconsistency among clinical studies may be due to the limited understanding of precise mechanisms by which estrogen affects the dopaminergic system in the brain. Estradiol was shown to be essential in maintaining the integrity of nigostratial pathways in rodents and in an acute rodent MPTP model of PD (20, 50). Estradiol has been demonstrated to exert antioxidant and neurotrophic properties, to modulate neuronal plasticity, and to decrease degeneration of dopaminergic neurons (51).

We acknowledge certain limitations in this study. To study the effect of reproductive factors on the age of PD onset, we performed a retrospective cohort study. Thus, we cannot rule out recall bias, e.g., regarding the disease onset or reproductive events such as age of menarche and menopause. However, it has been shown that recalling important reproductive events is very reliable and consistent (52). Furthermore, patient data from only one center with a Caucasian ethnicity were analyzed. For future studies, special attention should be paid on the effect of ethnicity on dopaminergic medication and gender differences.

Taken together, our study shows a strong association between reproductive factors and age of PD onset. A more detailed and in depth understanding of gender differences and the role of estrogen in PD may contribute to etiological conclusions and reveal novel treatment approaches.

## Ethics Statement

The study was approved by the Institutional Review Board for Ethics, FAU Erlangen-Nürnberg (152_15 Bc).

## Author Contributions

JW and JS designed the work; DF, GJ, JK, JW, and JS acquired data; OB and DF performed statistical analysis; DF, JW, and JS interpreted the data; DF and JS wrote the initial draft, and all authors revised the work critically for important intellectual content; all authors approved the final version of the manuscript and all authors agreed to be accountable for all aspects of the work in ensuring that questions related to the accuracy or integrity of any part of the work are appropriately investigated and resolved.

## Conflict of Interest Statement

The authors declare that the research was conducted in the absence of any commercial or financial relationships that could be construed as a potential conflict of interest.
