# Supplementary material for: Increase of Reproductive Life Span Delays Age of Onset of Parkinson’s Disease
Source: Front Neurol. 2017 Aug 21;8:397. doi: 10.3389/fneur.2017.00397 (PMC5566617; doi:10.3389/fneur.2017.00397)
Supplement: Supplementary file 1 [file Table_1.DOCX]

Supplementary Table

**Increase of reproductive life span delays age of onset in**

**Parkinson’s disease**

**Dominik Frentzel^1^, Grigorij Judanin^1^, Olga Borozdina^2^, Jochen Klucken^1^, Jürgen Winkler^1*^, Johannes CM Schlachetzki^1,3*^**

*** Correspondence:** Corresponding Authors: [Johannes.schlachetzki@uk-erlangen.de](mailto:Johannes.schlachetzki@uk-erlangen.de) &juergen.winkler@uk-erlangen.de

| **Supplementary Table 1**. Type of dopaminergic medication using matched-pairs design | | | |
| --- | --- | --- | --- |
| **Type of PD Medication** | **male** | **female** | **p-value ^a^** |
| L-Dopa only | 7 (9.0%) | 8 (10.3%) | 0.786 |
| L-Dopa + Other* | 12 (15.4%) | 13 (16.7%) | 0.827 |
| Dopamine agonist only | 6 (7.7%) | 1 (1.3%) | 0.053 |
| Dopamine agonist + Other* | 19 (24.4%) | 18 (23.1%) | 0.851 |
| L-Dopa + Dopamine agonist | 11 (14.1%) | 13 (16.7%) | 0.657 |
| Others* | 18 (23.1%) | 17 (21.8%) | 0.848 |
| * Others include: Amantadine, Rasagiline, Selegiline, Biperidene, Trihexyphenidyl, Bornaprine  ^a^ Chi-Square Test | | | |
